# Effect of mast cell stabilization on angiogenesis in primary and secondary experimental *Trichinella spiralis* infection

**DOI:** 10.1186/s13071-021-05075-9

**Published:** 2021-11-06

**Authors:** Marwa A. EL-Dardiry, Amany A. Abdel-Aal, Magda S. A. Abdeltawab, Mona El-Sherbini, Marwa A. Hassan, Asmaa A. Abdel-Aal, Manal Badawi, Shady E. Anis, Bahaa-Eldin A. Khaled, Abeer S. Al-Antably

**Affiliations:** 1 Department of Medical Parasitology, Faculty of Medicine, Fayoum University, Fayoum, Egypt; 2 Department of Medical Parasitology, Faculty of Medicine, Cairo University, Giza, Egypt; 3 Department of Postgraduate Studies & Scientific Research, Armed Forces College of Medicine (AFCM), Cairo, Egypt; 4 Department of Clinical Pathology, Faculty of Medicine, Cairo University, Giza, Egypt; 5 Department of Pathology, National Research Center, Giza, Egypt; 6 Department of Pathology, Faculty of Medicine, Cairo University, Giza, Egypt; 7 Department of Anatomy & Embryology, Faculty of Medicine, Cairo University, Giza, Egypt; 8 Department of Anatomy, Jouf University, Sakaka, Saudi Arabia

**Keywords:** *T. spiralis*, Mast cells, Ketotifen, Albendazole, Angiogenesis, VEGF

## Abstract

**Background:**

Mast cells are known to affect the primary and secondary immune responses against parasites, and this effect is partially mediated through the release of pro-angiogenic mediators. The aim of this study was to explore the effect of the mast cell stabilizer (MCS), ketotifen, with and without albendazole, an anti-parasitic prescription medicine, on the inflammatory response against *Trichinella spiralis*, with the overall aim to investigate its effect on angiogenesis accompanying nurse cell formation.

**Methods:**

The effect of ketotifen and albendazole was explored in eight groups of female BALB/c mice. Four groups were sensitized with a small dose of* T. spiralis* larvae. The drug regimen was then applied to both sensitized (challenged) and non-sensitized mice. The parasite load was assessed by histopathological examination of the small intestine and muscle tissue, and angiogenesis was assessed by immunohistochemistry to determine the expression of vascular endothelial growth factor (VEGF).

**Results:**

Sensitized mice showed a significantly lower parasite load and a more pronounced inflammatory response than mice receiving a single infective dose of *T. spiralis* larvae. All treated groups showed a significant reduction in parasite count compared to the control groups (groups IAa and IBa), reaching approximately an 98.8% reduction in adult parasite count in the sensitized group treated with albendazole (groups IIAb and IIBb). MCS significantly decreased the parasite count during both the intestinal or muscular phases, reduced tissue inflammation, and decreased local VEGF expression, both in the non-sensitized and sensitized groups.

**Conclusion:**

Sensitization with a low dose of *T. spiralis* larvae was found to confer a partial protective immunity against re-infection and to positively affect the study outcomes, thus underlining the importance of vaccination, but after extensive studies. The anti-angiogenic effect of MCS protects against larval encystation during the muscle phase. The anti-angiogenic potential of albendazole suggests that the action of this anti-helminthic during trichinellosis is not confined to structural damage to the parasite cuticle but includes an effect on host immunopathological response.

**Graphical Abstract:**

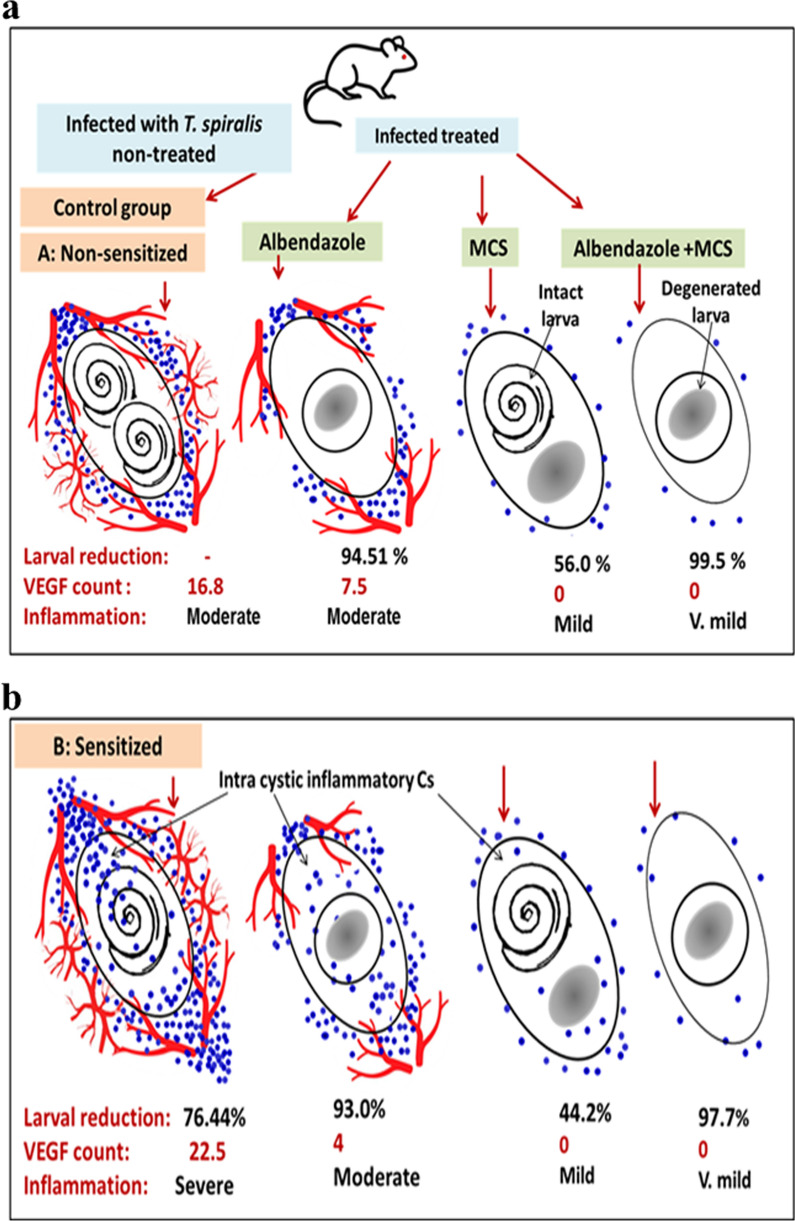

## Background

*Trichinella* infection in humans and animals results from the ingestion of first-stage larvae in uncooked or undercooked meat, with the most notorious sources being pork and the meat of animals such as bears. *Trichinella spiralis* was the first species to be discovered [10], and it is also the most virulent and pathogenic species to humans. Its higher pathogenicity, compared to that of other* Trichinella* species, is due to the large number of larvae produced by the gravid female and the magnitude of the immune response to different stages of the parasite [20]. *Trichinella* infection in all affected hosts, including humans, passes through two phases: the intestinal phase and the muscular phase. Subsequent complications, such as cardiac or neurological problems, result from the aggressive pathogenesis caused by the larval stages and may lead to a fatal outcome [10, 20].

Benzimidazoles, such as albendazole and mebendazole, are the most common anti-helminthics used in the management of *T. spiralis* infection. Albendazole has an advantage over mebendazole as its recommended plasma levels are achieved in most patients, so it does not require any monitoring [10]. Both drugs are characterized by a limited bioavailability, a high degree of resistance, and weak action against encysted larvae [7]. In addition to its anti-helminthic activity, albendazole has been found to have an anti-angiogenic effect in both cancerous and non-cancerous models of angiogenesis. These effects are mediated, partially, by through a decrease in the level of vascular endothelial growth factor (VEGF) and the downregulation of VEGF-2 receptors [22]. VEGF plays an important role in the pathological process of angiogenesis during inflammation or cancer [26].

VEGF is upregulated during *T. spiralis* infection to provide the necessary nourishment and maintain *T. spiralis* larval stages within affected muscles [15]. Pro-angiogenic factors are secreted by a variety of immune cells, including mast cells. These factors play a key role in the termination of the intestinal phase, and thus the infection, by helping the host expel adult worms within 2 weeks of infection by developing what is called “intestinal mastocytosis” [5, 18, 28]. It was previously reported that the persistence of *T. spiralis* infection results from the late emergence of intestinal mast cells and the related inflammation [1]. Immunization has been reported to accelerate rapid expulsion through augmenting the intestinal mast cell population [3]. Therefore, hypothetically, mast cell stabilizers may negatively affect expulsion in both primary and challenge infection and thus support the maintenance of *T. spiralis* infection.

Mast cell stabilizers (MCSs) are common medications that are known to decrease the degranulation of mast cells, thus preventing the release of histamine and other chemotactic factors [27]. Ketotifen is a MCS that also has antihistamine properties and is used in the treatment of a wide range of allergic and inflammatory disorders [2].

The present study explores the effect of the MCS ketotifen on the inflammatory response during the intestinal and muscular phases related to both primary and secondary experimental *T. spiralis* infection. We also investigated the effect of ketotifen on albendazole and the process of angiogenesis accompanying the nurse cell formatio using VEGF as a marker for angiogenesis.

## Methods

### Ethical considerations

The current study was conducted according to the national and international guidelines on animal research and followed the strict quality measures of the Animal Center of the Theodor Bilharz Research Institute (TBRI) and guidelines of Animal Research: Reporting of In Vivo Experiments (ARRIVE) (https://arriveguidelines.org/).

### Experimental animals and infection

A total of 56 female BALB/c mice, aged 6 to 8 weeks and weighing 25–30 g each, were obtained from the Animal Center of the TBRI. The animals were housed in accordance with the well-organized rearing TBRI laboratory system at a temperature of 22 ± 2 °C, an accurate light/dark photocycle, and relative humidity of about 55%. The *T. spiralis* strain used for the infection was obtained from the same animal center. Non-sensitized mice were infected orally with 200 ± 10 larvae/mouse. The process of animal infection was initiated by mincing pieces of heavily infected diaphragms, followed by digestion in 1% pepsin-hydrochloride, and then incubation overnight at 37 °C. Using the sedimentation technique, larvae were collected, washed in physiological saline (0.85%) 4 times, counted, and then stored for further usage in oral infection [9].

### Sensitization

A small dose infection strategy was used to sensitize the mice [19]. Initial primary infection was obtained by infecting the animals orally with 10 larvae, followed infection with another 10 larvae after 1 week. Then, after another 2 weeks, challenge infection was completed by infection the mice with an oral *T. spiralis* dose of 200 ± 10 larvae/mouse. Similarly, non-sensitized mice were infected with doses of 200 ± 10 *T. spiralis* larvae/mouse (primary infection).

### Study groups

Infected mice were divided into sensitized (I) and non-sensitized (II) groups. Each group was subdivided into two other groups, the intestinal (A) and the muscular phases (B). These groups were further subdivided into four subgroups (a, b, c, and d), with each subgroup including seven mice, as follows:

- Groups IAa and IBa: non-sensitized, infected, non-treated (NS control groups [NS-C]).
- Groups IAb and IBb: non-sensitized, infected, treated with albendazole only (NS-ALB).
- Groups IAc and IBc: non-sensitized, infected, treated with MCS only (NS-MCS).
- Groups IAd, and IBd: non-sensitized, infected, treated with albendazole and MCS (NS-ALB + MCS).
- Groups IIAa and IIBa: sensitized, infected, non-treated (S control groups [S-C]).
- Groups IIAb and IIBb: sensitized, infected, treated with albendazole only (S-ALB).
- Groups IIAc and IIBc: sensitized, infected, treated with MCS only (S-MCS).
- Groups IIAd and IIBd: sensitized, infected, treated with albendazole and MCS (S-ALB + MCS).

### Medicinal agents

Albendazole (Bendax) was purchased and prepared as a 20 mg/ml suspension (Sigma Pharmaceutical Industries, Menofia, Egypt). A dose (50 mg/kg) of albendazole was given orally from the second day post-infection (dpi) for the following 3 days. Following Zhu et al. [32], pure powder of ketotifen was dissolved in carboxymethylcellulose sodium 0.5% w/v in saline and given orally (1 mg/kg/day). Mice were treated with MCS 2 days pre-infection and then once daily until the end of the experimental study. At the end of experiments, the mice in all groups were anesthetized with 5% of isoflurane vapor until sedation and then euthanized by cervical dislocation [13]. Muscles and intestine were collected from all mice.

### Parasitological analysis

The parasite load was calculated by counting the number of adult worms in the intestine on 7 dpi and the number of larvae in the muscles on 40 dpi. The parasite load during the intestinal phase of infection was determined by counting adult parasites according to Wakelin and Lloyd [31]. Mice were euthanized on 7 dpi, following which the small intestines were isolated and washed in physiological saline. The specimens were then dissected into 1-cm segments and the segments incubated in physiological saline for 2 h at 37 °C. The segments were then washed three times and the supernatant from each wash was collected and centrifuged at 2000 rpm for 3 min. The number of adults in the sediment was counted under the inverted microscope at low-power magnification (×100) and calculated per 100 ml intestinal fluid. Similarly, larvae in the muscle specimens were counted according to Dunn and Wright [9].

### Histopathological examination

To study the effect of the above treatments on the intestinal phase, we used a 1-cm piece of tissue from the small intestine, taken at the junction of the proximal one-third and distal two-thirds, from each mouse that was sacrificed on 7 dpi. For the muscle phase, skeletal muscle specimens from the diaphragm and the hind legs were isolated from mice that were sacrificed on 40 dpi. All specimens were fixed in 10% formalin, dehydrated, and embedded in paraffin blocks; the blocks were sliced into 5-µm-thick sections, and the sections stained by hematoxylin and eosin and examined under the microscopic at ×100 magnification.

### Immunohistochemistry and image analysis

Tissue sections (5 μm thick) were deparaffinized in xylene and then rehydrated through a descending series of alcohol. These sections were then incubated in 3% hydrogen peroxide for 5 min to block endogenous peroxidase activity, followed by two washes of 5 min each in phosphate-buffered saline (PBS). The tissue sections were then immersed in 0.01 mol/l citrate buffer (pH 6) in a water bath for antigen retrieval, followed by incubation with the primary antibody murine anti-human VEGF monoclonal antibodies (Dako North America Inc., Carpinteria, CA, USA) for 1 h at room temperature. Later, the sections were washed three times in PBS for 15 min. Biotinylated goat anti-polyvalent secondary antibody and streptavidin peroxidase enzyme were added to the tissue samples for 10 min, followed by another wash in PBS. Peroxidase activity was visualized by the chromogenic substrate for peroxidase, denzymediaminobenzidine (DAB), as described by Ramos-Vara and Miller [23]. Hematoxylin counterstain was used after washing the sections, followed by dehydration. These tissue sections were then cleared in xylene and mounted on slides with DPX. A brownish immunostaining for VEGF was considered to be positive, in comparison to the gray coloration of negative samples. The internal negative and positive control samples were provided within the kit to validate the results. To quantitatively estimate the local expression of VEGF, a specific analysis was performed using the Leica Qwin 500 Image Analyzer (LEICA Imaging Systems Ltd., Cambridge, UK). Optical density and area percentage were automatically calculated in 10 fields on a real-time image from the microscope that was connected to a video monitor.

### Statistical analysis

The statistical significance was evaluated at *P* < 0.05. One-way analysis of variance was applied to compare the means and standard deviations of the different study groups. The Tukey–Kramer post-hoc test was used to determine the mean differences and statistical significances between the results of the various treatment groups.

## Results

### Sensitization decreased parasite load during subsequent *T. spiralis* infection during both the intestinal and muscular phases

**Fig. 1:**
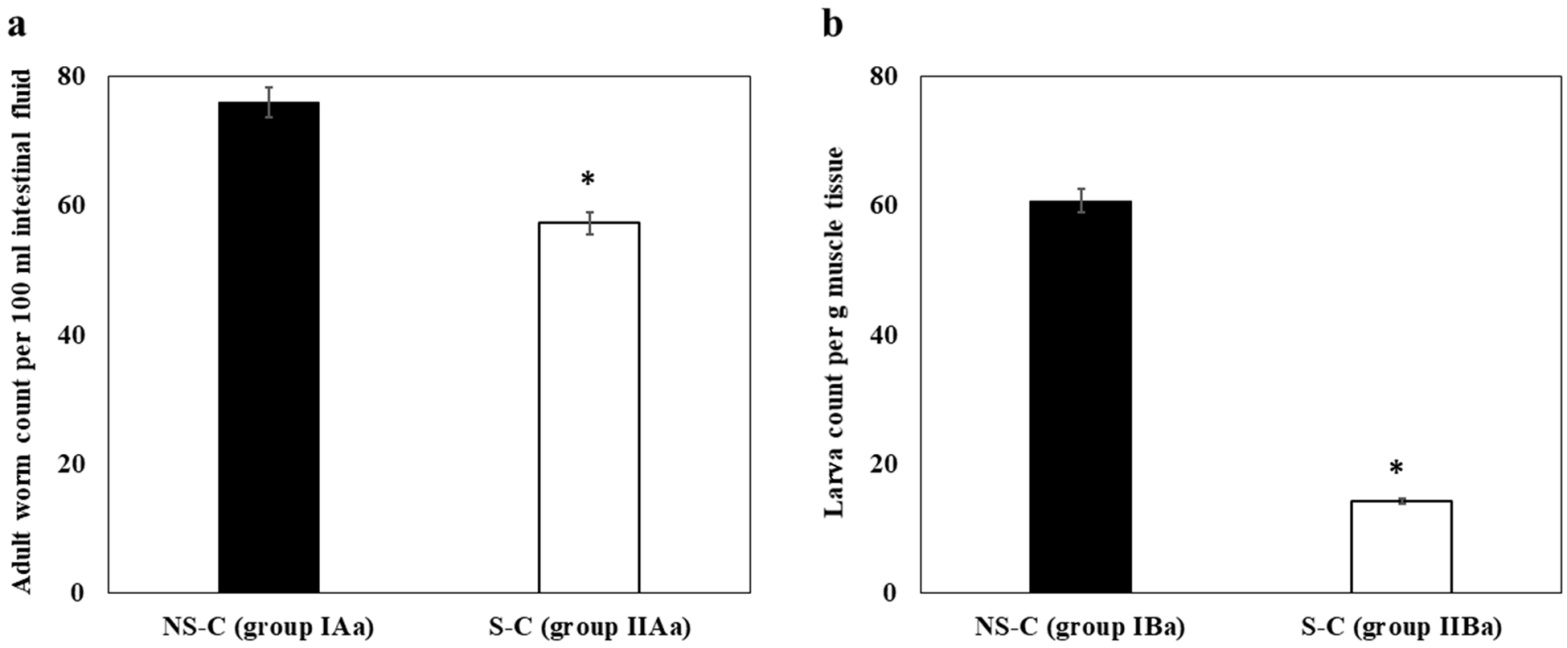
Bar graph showing means and standard deviations (SD) of parasite count (*y*-axis) in NS-C (groups IAa and IBa) and S-C (groups IIAa and IIBa) mice (*x*-axis). **a** Adult worm count per 100 ml intestinal fluid, **b** larval count per gram muscle tissue. Asterisk indicates a significant difference at *P* < 0.05 (one-way analysis of variance [ANOVA]). Abbreviations: NS-C, Control non-sensitized mice; S-C, sensitized, infected, non-treated mice

In infected non-sensitized and non-treated mice (the NS-control group; gGroup IAa), the adult intestinal worm count was 76.0 ± 3.12/100 ml intestinal fluid. In comparison, in the sensitized control group (group IIAa), the count was significantly reduced by 24.6% (57.3 ± 2.18/100 ml) (Fig. 1a). The reduction in parasite count was even more pronounced during the muscular stage of infection (by 76.44%), where the larval count in the sensitized group (Group IIBa) was 14.3 ± 2.65/g muscle tissue as compared to 60.7 ± 7.05/g in the non-sensitized group (group IBa) (Fig. 1b).

### MCS reduced intestinal worm burden more efficiently in sensitized mice than in non-sensitized mice, while in the muscle phase, it was more effective in reducing the larval load in non-sensitized mice

In non-sensitized mice receiving MCS only (group IAc), the adult intestinal count was 56.7 ± 6.61/100 ml intestinal fluid, which is a reduction of 25.39% compared to the control non-sensitized group. Sensitized mice receiving MCS only had an intestinal count of 38.7 ± 6.14/100 ml, which is a 32.46% reduction compared to the control sensitized group (group IIAa).

During the muscle phase, the non-sensitized group that received MCS only (group IBc) had a larval count of 26.7 ± 1.32/g muscle tissue, which is a 56.01% reduction compared to the larval count in the control non-sensitized group (group IBa). In comparison, sensitized mice receiving MCS only (group IIBc) had a larval count of 8.0 ± 0.87/g, which is a 44.06% reduction compared to the control sensitized group (group IIBa).

### The addition of MCS to albendazole did not significantly improve the reduction in parasite load achieved by albendazole alone

**Fig. 2:**
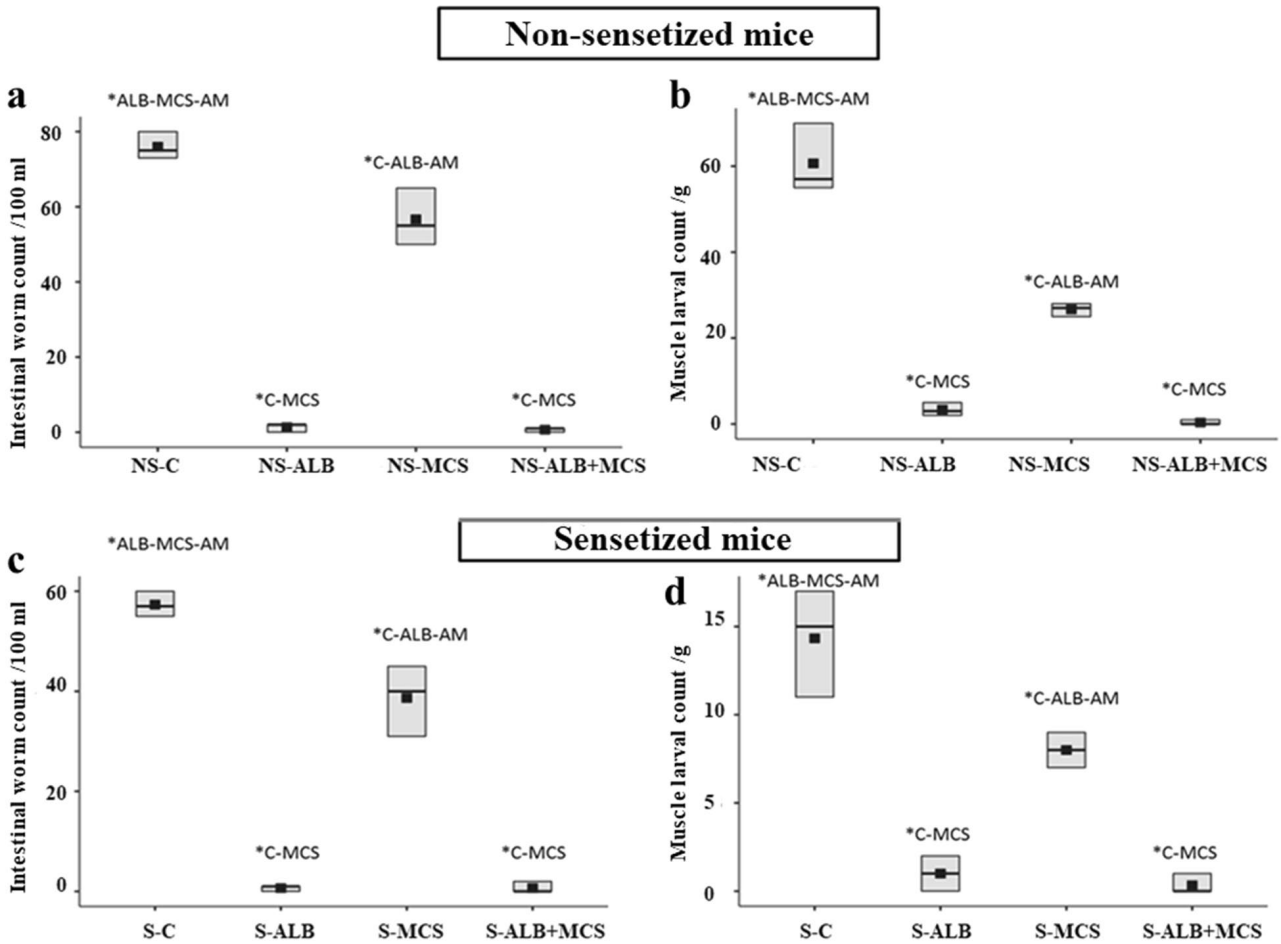
Boxplot comparing the effect of different drug regimens showing the means and interquartile ranges of parasite load in both non-sensitized and sensitized mice. **a** Intestinal worm count/100 m intestinal fluid in non-sensitized mice. **b** intestinal worm count/100 ml intestinal fluid in sensitized mice, **c** muscle larval count/g muscle tissue in non-sensitized mice, **d** muscle larval count/g muscle tissue in sensitized mice. Asterisk indicates significant difference at *P* < 0.05 (one-way ANOVA followed by Tukey–Kramer post-hoc comparison test). Abbreviations: ALB, Albendazole monotherapy group; AM, combined albendazole and MCS group; C, control infected non-treated mice; MCS, mast cell stabilizer monotherapy group; NS, Non-sensitized mice; S, sensitized mice

The administration of albendazole led to a significant decrease in the number of adult worms in both sensitized and non-sensitized mice. The adult worm count in non-sensitized mice treated with albendazole (group IAb) was 1.33 ± 1/100 ml intestinal fluid, which is a 98.25% reduction from the control non-sensitized group (group IAa) (Fig. 2a). In the sensitized group (group IIAa), the adult intestinal count in albendazole-treated mice was 0.67 ± 0.50/100 ml, reflecting a reduction of 98.8% compared to control sensitized mice (group IIAa). In non-sensitized mice receiving both albendazole and MCS (group IAd), the adult intestinal count was 0.67 ± 0.50/100 ml. In sensitized mice receiving both drugs (group IIAd), the adult worm count was 0.67 ± 0.50/100 ml (Fig. 2b). In both non-sensitized and sensitized groups, the effect produced by the administration of both drugs was not significantly different from the effect produced by albendazole alone (*P* = 1.0 in both groups).

During the muscle phase, albendazole treatment significantly decreased the larval count in both sensitized and non-sensitized mice. The number of larvae in non-sensitized mice treated with albendazole (group IBb) was 3.33 ± 1.32/g muscle tissue, constituting a 94.51% reduction from the larval count in the control non-sensitized group (group IBa) (Fig. 2c). In the sensitized group (group IIBb), the encysted larval count in albendazole-treated mice was 1.0 ± 0.87/g, which is a reduction of 93.01% compared to the control sensitized mice (group IBa) (Fig. 2d). The larval count in mice receiving both albendazole and MCS was 0.33 ± 0.5/g in both sensitized and non-sensitized mice. In both non-sensitized and sensitized groups, the effect produced by the administration of both drugs was not significantly different from the effect produced by albendazole alone (*P* = 0.32 and *P* = 1.0, respectively).

### Effect of different drug regimens on the local inflammatory response

#### Intestinal phase

**Fig. 3:**
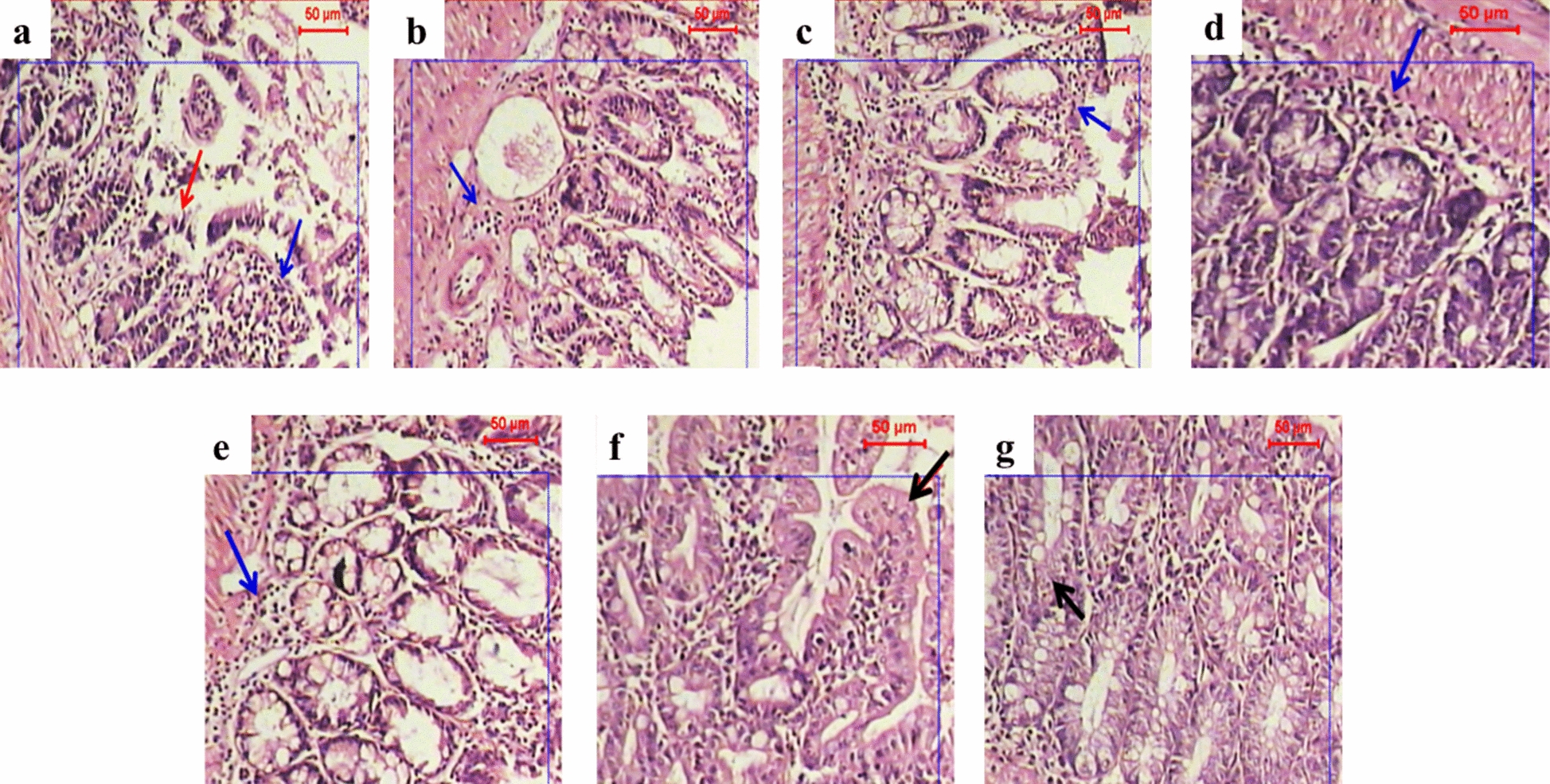
Photographs of intestinal sections from different treatment groups. Staining: hematoxylin and eosin (H&E); magnification: ×100. Blue arrows indicate inflammatory cells rich in eosinophils, red arrows indicate damaged mucosa, and black arrows point to the restoration of intestinal villi. **a** Disrupted fragmented intestinal mucosa of infected, non-treated mice showing numerous accumulations of inflammatory cells.** b**,** c** Sensitized (**b**) (groupIIAb) and non-sensitized (**c**) (group IAb) mice treated with albendazole, showing a moderately improved intestinal epithelium with abundant inflammatory cells. **d**, **e** Sensitized (group IIAc) (**d**) and non-sensitized (**e**) (group IAc) mice treated with MCS, showing mild inflammatory reaction and moderately disturbed intestinal epithelium. **f**,** g** Sensitized (**f**) (group IIAd) and non-sensitized (**g**) (group IAd) mice treated with combined therapy, showing very mild inflammatory reaction and an almost healthy intestinal epithelium

The intestinal tissue sections from infected, non-treated mice showed fragmentation and dense eosinophilic infiltration (Fig. 3a). The isolated intestinal specimens from albendazole-treated mice either sensitized or not showed a moderate improvement in intestinal pathology with persistent eosinophilic infiltration (Fig. 3b, c). The specimens derived from mice treated with MCS (sensitized or non-sensitized) displayed mild inflammation and moderate disturbance of the intestinal epithelium (Fig. 3d, e). The tissue sections from mice in the combined regimen groups (receiving albendazole and MCS), both sensitized and non-sensitized, showed mild inflammation and an almost healthy intestinal epithelium (Fig. 3f, g).

#### Muscle phase

**Fig. 4:**
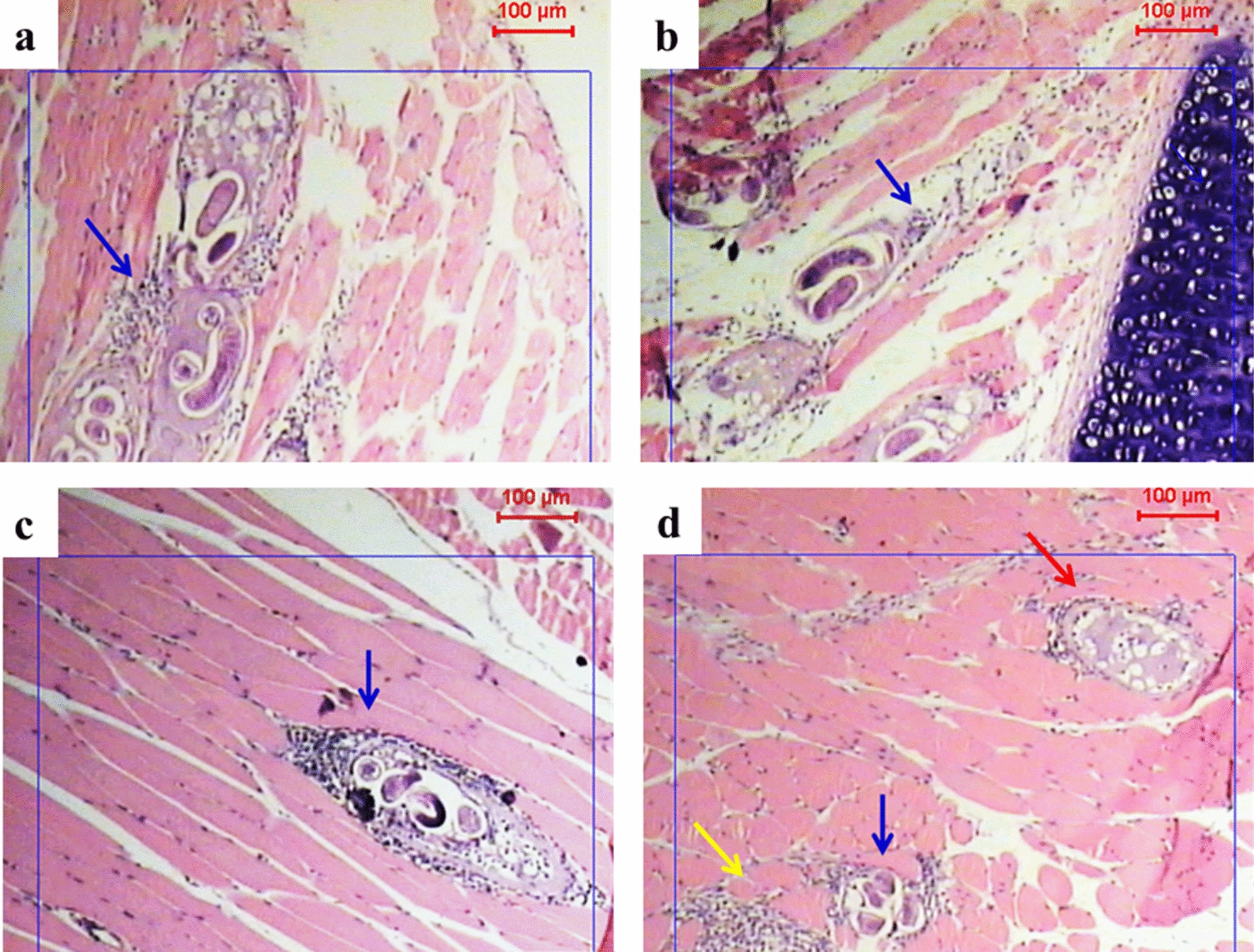
Photographs showing infected muscle tissues with *T. spiralis* larvae within the two control groups (groups IBa and IIBa). Staining: H&E; magnification: ×50. Blue arrows indicate inflammatory cells, red arrow indicates hyaline wall, and yellow arrow points to fragmented larvae. **a**, **b** Non-sensitized mice (group IBa), showing intact larvae inside cystic lesions surrounded by a moderate number of inflammatory cells. **c**, **d** Sensitized mice (group IIBa), showing intense inflammatory response with intact larval stages (**c**) and fragmented larvae or/and replacement of larvae by a hyaline material (**d**)

Muscle sections from control, infected, non-sensitized mice (Fig. 4a, b) showed intact larval stages inside several cystic lesions surrounded by moderate inflammation with the absence of inflammatory cells inside the cystic lesion.

Muscle sections from mice in the sensitized, control groups (Fig. 4c, d) showed a relatively intense inflammatory response, expressed by areas rich in eosinophils. Some intact larvae were seen, while others were fragmented and surrounded by an intense inflammatory reaction.

**Fig. 5:**
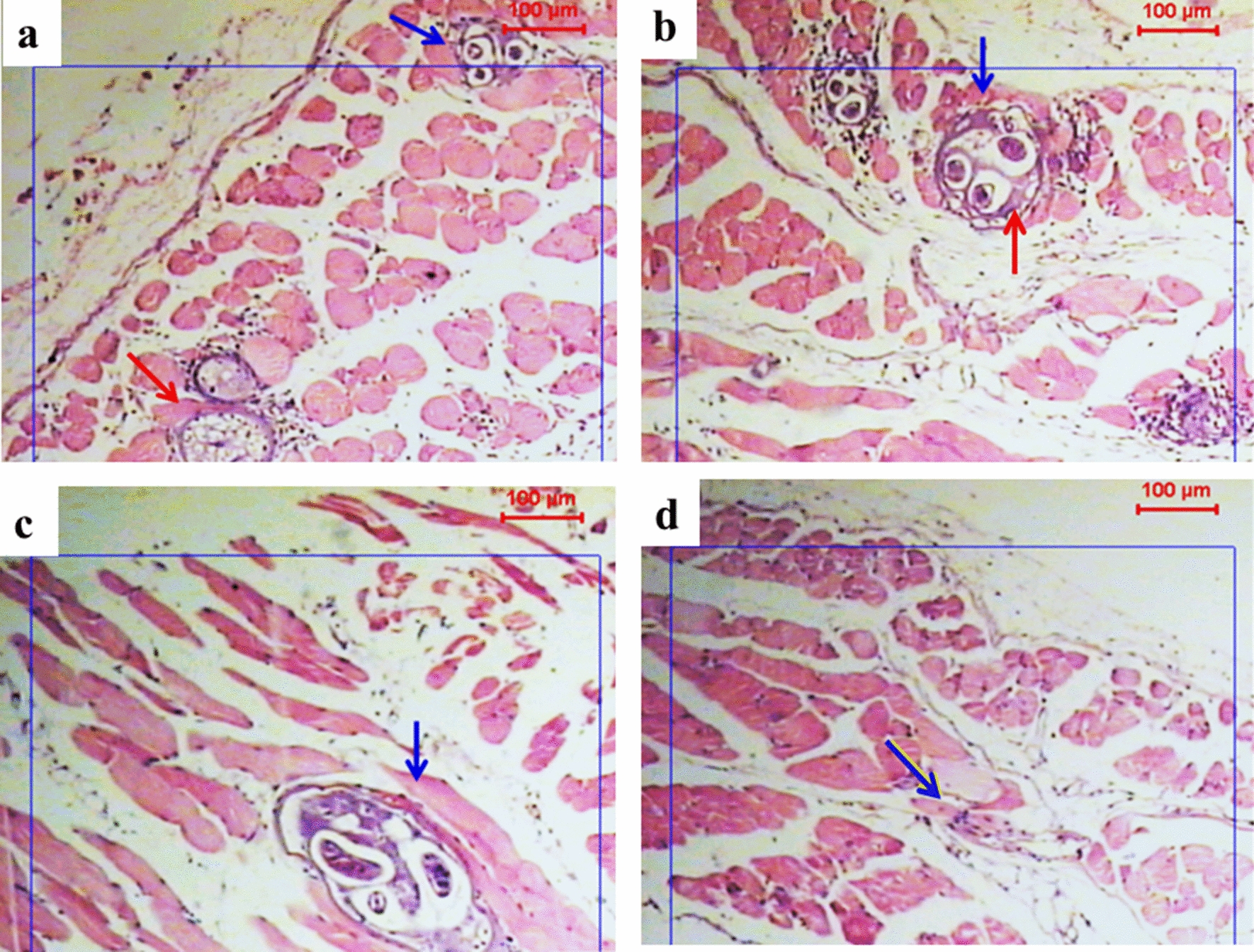
Photographs showing infected muscle tissues with *T. spiralis* larvae in two groups treated with MCS only (groups IBc and IIBc). Staining: H&E; magnification: ×50. Blue arrows indicate inflammatory cells, red arrows show vacuolation or/and hyaline wall. **a**, **b** Non-sensitized mice treated with MCS (group IBc); some larvae are intact and some show features of degeneration, such as vacuolation or/and hyaline appearance with very few inflammatory cells. **c**, **d** Sensitized mice (group IIBc) with a few cystic lesions, with intact larva (**c**) and those showing absence of cystic lesions with a few inflammatory cells (**d**)

Sections from non-sensitized mice treated with MCS monotherapy (group IBc) showed both intact and degenerated larvae, with few inflammatory cells around the cystic lesions (Fig. 5a, b). Sensitized mice treated with MCS (group IIBc) only showed few cystic lesions, some intact larval stages, and areas with a complete absence of cystic larval lesions with few inflammatory cells, mainly monocytes (Fig. 5c, d).

**Fig. 6:**
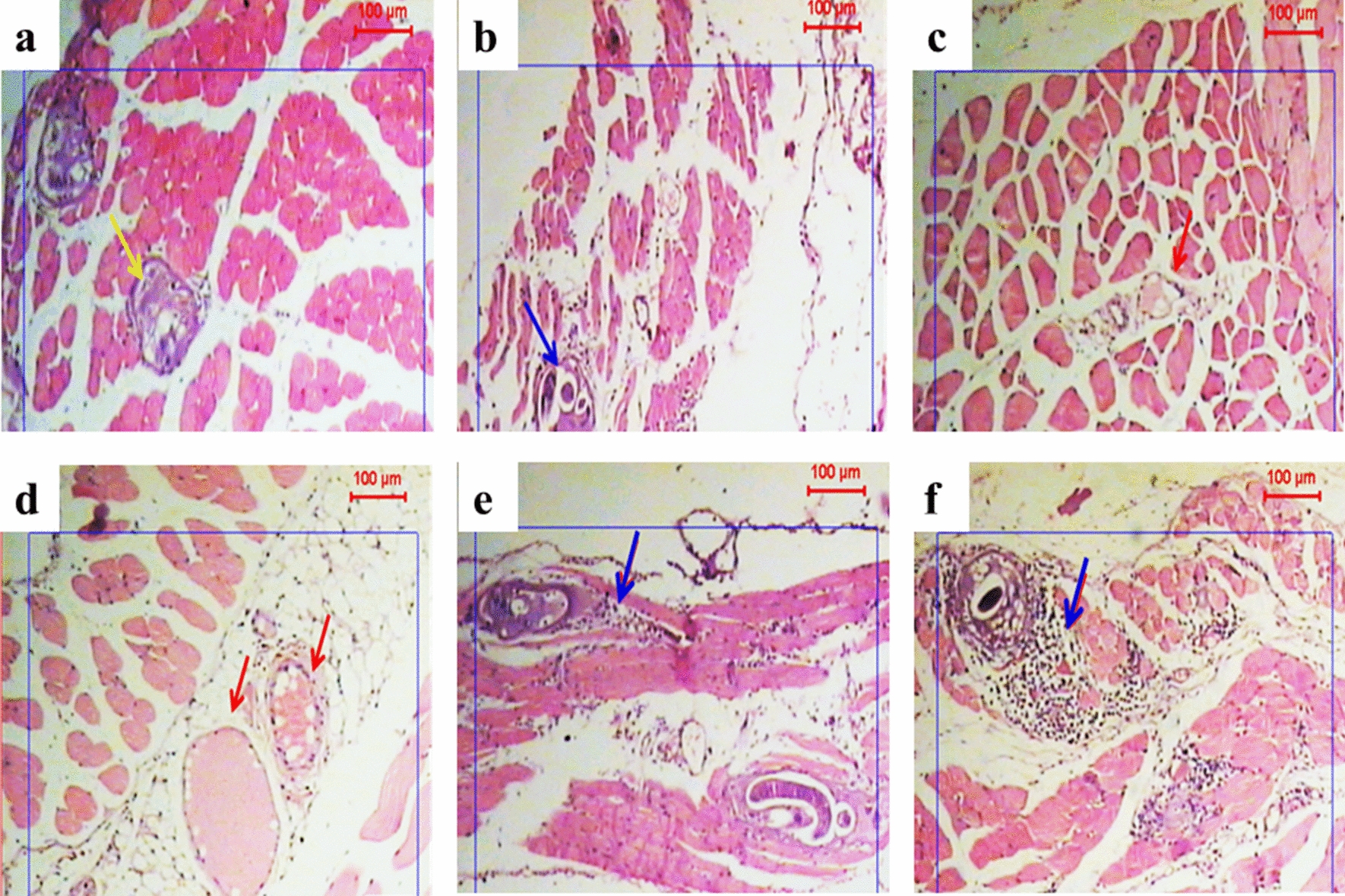
Photographs showing infected muscle tissues with *T. spiralis* larvae in two treated groups using combined therapy (ALB + MCS) (groups IBd and IIBd) (**a**–**d**) and those treated with albendazole (groups IBb and IIBb) (**e**, **f**). Staining: H&E; magnification: ×50. Blue arrows indicate inflammatory cells, red arrows show vacuolation or/and hyaline wall, and yellow arrow points to fragmented larvae. **a**, **b** Non-sensitized mice (group IBd); some larvae show features of degeneration (**a**) and some are seen to be intact (in **b**), and both are surrounded by mild inflammatory response. Best results are seen in **c** and **d**, representing sensitized mice (group IIBd) with a complete absence of the cystic larval stage with very few inflammatory cells. **e** Non-sensitized mice (group IBb); larvae are intact or show features of degeneration and surrounded by moderate inflammatory reaction. **f** Sensitized mice (group IIBb); a larval stage shows features of degeneration surrounded by intense inflammatory response

Sections from non-sensitized mice receiving both albendazole and MCS (group IBd) showed some larvae with features of degeneration and other larvae that were intact (Fig. 6a, b). The muscle tissue specimens displayed a relatively mild inflammatory response composed mainly of monocytes, accompanied with an improvement of the pathology and complete absence of larval encystation (Fig. 6c, d). Sections from non-sensitized mice treated with albendazole (group IBb) showed larval degeneration and moderate inflammation (Fig. 6e). Sections from sensitized mice receiving albendazole (group IIBb) showed larval degeneration surrounded by an intense inflammatory cellular infiltration, composed mainly of eosinophils (Fig. 6f).

### Effect of different drug regimens on VEGF expression

**Fig. 7:**
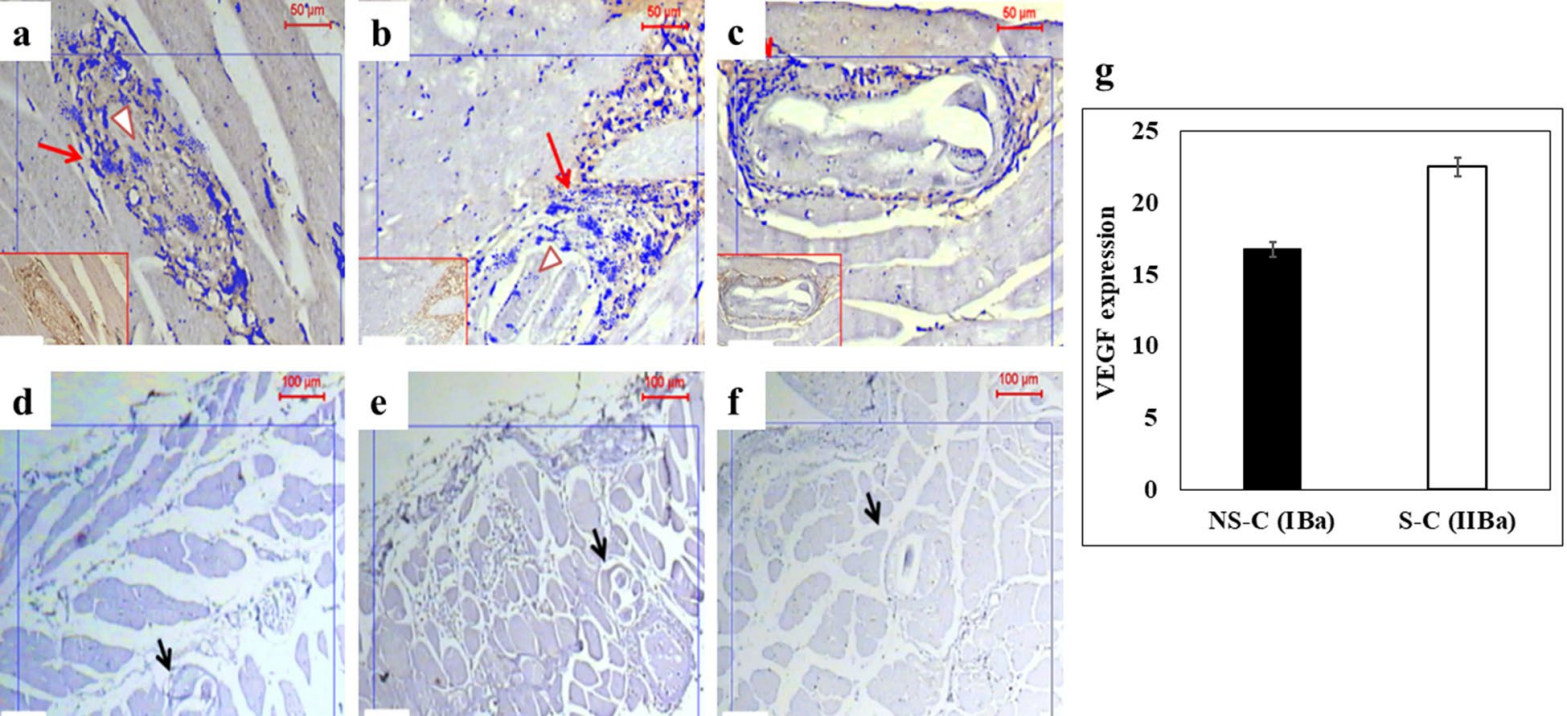
Photographs of muscular tissue sections showing the results of immunohistochemical staining specific for VEGF. Magnification: ×100. Red arrows indicate positive VEGF expression around the cystic lesion and within the lesions (arrowheads), and black arrows point to degenerated larvae. **a** Positive expression for VEGF, within and around cysts in the control group of non-sensitized, infected, not treated mice (group IBa). **b** Positive expression for VEGF in sensitized, infected, not treated mice (group IIBa). **c** Expression of VEGF in groups treated with albendazole with only minimal expression around the cyst and almost complete absence of the protein within the cyst. **d**–**f** Negative expression in all groups treated with MCS; non-sensitized mice treated with MCS (Group IBc) (**d**), sensitized mice treated with MCS (group IIBc) (**e**) and mice treated with combined therapy (**f**). **g** Mean expression levels of VEGF in skeletal muscle tissue (*y*-axis) of infected, control, sensitized and non-sensitized mice (*x*-axis). Significant difference at *P* < 0.05 (one-way ANOVA). Abbreviations: VEGF, Vascular endothelial growth factor

Local expression of VEGF was detected in control non-sensitized mice (groups IAa and IBa) (16.8 ± 1.58/g muscle tissue). Sensitized mice (groups IIAa and IIBa) showed a higher expression of VEGF (22.5 ± 2.88/g). The administration of albendazole led to a significant reduction in VEGF levels in both non-sensitized (7.5 ± 1.2/g) and sensitized mice (4.0 ± 0.76/g). Both of the non-sensitized and sensitized mice receiving MCS with or without albendazole did not show any expression of VEGF in infected muscle fibers (Fig. 7; Table 1).

**Table 1:** Vascular endothelial growth factor expression levels in skeletal muscle sections from the different study groups

Study groups^a^	Vascular endothelial growth factor expression in skeletal muscle tissue			
	Non-sensitized mice		Sensitized mice	
	Mean	Standard deviation	Mean	Standard deviation
Infected control groups (groups IBa and IIBa)	16.75	1.58	22.50	2.88
Albendazole groups (groups IBb and IIBb)	7.50*	1.20*	4.00*	0.76*
MCS groups (groups IBc and IIBc)	0.00*	0.00*	0.00*	0.00*
Combined albendazole and MCS groups (groups IBd and IIBd)	0.00*	0.00*	0.00*	0.00*

*MCS* Mast cell stabilizer

*Significant difference to control at *P* < 0.05 (one-way analysis of variance)

^a^See “Study groups” section for complete description of treatments

## Discussion

The immune response to trichinellosis is a complex interplay of innate and acquired humoral and cellular immune responses. Many immune effector cells play a dual role in protection and immunopathology during infection with *Trichinella* spp. The most notable immune cells playing a pivotal role during the primary and secondary infection with *T. spiralis* are eosinophils and mast cells [6].

In our study, primary infection with a low infective dose of 10 *T. spiralis* larvae resulted in partial protection against re-infection, as reflected by the significant decrease in the counts of adult worms during the intestinal phase and in larvae during the muscle phase. This protective effect was more pronounced during the muscle phase of infection, during which the reduction in larval count reached 76.44% in sensitized mice; in comparison, the reduction in intestinal worm count was 24.6% during the intestinal phase. Protection against secondary *Trichinella* infection is dose-dependent, as demonstrated by Murrell [19] who reported that infection with a low dose of *T. spiralis* larvae resulted in a boost of the immune response. Subsequent infection using variable doses (112, 500, 10,000, and 25,000 larvae, respectively) resulted in resistance to re-infection. Protection against re-infection is possibly mediated through mast cells, eosinophils, mucosal immunity, and humoral immunity [4]. Doligalska [8] stated that infection with *T. spiralis* in mice generates an anti-inflammatory Th-2-mediated response, which controls the effector mechanism operating in the intestine and is associated with pronounced intestinal mastocytosis, eosinophilia, and destruction of the intestinal epithelial layer during the expulsion of parasites from the gut. This author further stated that protection is dependent on a non-specific inflammatory reaction mediated by mast cells. Doligalska [8] used ketotifen as an anti-allergic compound and observed a greater worm burden, which is not in agreement with our results. This result led Doligalska [8] to conclude that interaction between effector leukocytes and antibodies was not effective, leading to her proposing that other mechanisms, not related to hypersensitivity or conventional inflammatory response, regulated the level of infection.

In our study, repeated infection with *T. spiralis* larvae after sensitization with a low dose infection resulted in a more pronounced immune response with prominent eosinophilic infiltration. Drug treatment, whether with albendazole or with MCS, resulted in a partial reduction of inflammation and improvement of tissue structure. The eosinophilic response is an established feature of parasitic diseases, including trichinosis, which explains the increased co-incidence of allergic manifestations [6]. Studies on mice deficient in interleukin (IL) 5 have shown that the role of eosinophils in the expulsion of adult *T. spiralis* is more prominent during secondary rather than during primary infection [29, 30]. In contrast, eosinophils have been shown to exert a cytotoxic effect on *T. spiralis* during primary infection by antibody-dependent cellular cytotoxicity [11]. Interestingly, eosinophils are known to play a supportive role in larval growth through the IL-4/STAT6 signaling pathways, which results in a suppressed local inflammation and enhanced larval nutrition and metabolism [14].

While ketotifen is well known for its action as a MSC, it is also reported to affect eosinophils by interferring with eosinophil chemotaxis, impeding cellular functions, and inducing eosinophil necrosis [12, 16]. A few studies have reported the inhibitory effect of ketotifen on mucosal mast cell hyperplasia and intestinal dysmotility of rats infected with *T. spiralis* [8, 25]. These interactions, together with its mast cell stabilizing effect, may contribute to alleviation of the inflammatory response in parasitic diseases, as demonstrated in the current study.

During the muscle phase, *T. spiralis* larvae settle in skeletal muscle fibers by transforming themselves into nurse cells which become encysted inside a collagen capsule. The resulting complex *Trichinella* larvae nests require a growing supply of oxygen and nutrients, which is provided by the formation of new blood vessels. *Trichinella spiralis* is reported to induce angiogenesis through the induction of VEGF [21].

In the current study, infection with *T. spiralis* was found to be associated with a positive local expression of VEGF in skeletal muscle tissue in the non-sensitized, infected, non-treated group. The sensitized group showed significantly increased VEGF expression. A decrease in tissue expression of VEGF was observed in the albendazole-treated mice, while mice treated with MCS showed a complete absence of positive VEGF staining. Consistent with our findings, Kang et al. [15] reported that VEGF was expressed in muscle fibers of *T. spiralis*-infected mice, which provides the necessary blood supply to nourish and maintain *T. spiralis* larval stages within affected muscles. Mast cells are known to promote angiogenesis [17], which explains the increased expression of VEGF in sensitized as compared to non-sensitized mice, as demonstrated in the current study. The effect of ketotifen on granuloma size and new vessel formation was studied by Russo et al. [24] in Wistar rats. These authors observed a reduction in granuloma size and decreased angiogenesis, evidenced by a lower hemoglobin content, and decreased levels of tumor necrosis factor alpha. In the current study, decreased expression of VEGF was also observed in albendazole monotherapy. Pourgholami et al. [22] reported that an acute dose of albendazole demonstrated an anti-angiogenic effect by suppressing both hypoxia-inducible factors and VEGF.

## Conclusion

In conclusion, sensitization with a low dose of *T. spiralis* larvae conferred a partial protective immunity against re-infection. Prevention of mast cell degranulation, through the administration of ketotifen, reduced the parasite load and minimized the inflammatory reaction. The hypothesis of this research relied on the possibility of ketotifen, as a MCS, to negatively affect the desired outcomes of the secondary infection, which was expected to eliminate the adult parasites by removing intestinal mastocytosis, thus decreasing the number of larvae within the muscles. However, this was not the case in this study. Instead, groups receiving MCS were found to have significantly lower larval counts. To infer the reason behind such results, we explored the angiogenic process needed for nurse cell formation and found that the groups treated with MCS showed a 100% reduction in VEGF expression. Decreased angiogenesis can, partially, explain the reduction in muscle larval load since new vessel formation is an essential process in the formation of nest cells that nourish and support encysting larvae.

The anti-angiogenic effect of ketotifen also provides a protective effect against larval encystation during the muscle phase. As such, it can be of value as an adjuvant drug in conventional anti-helminthic therapy in the management of *T. spiralis* infection. The anti-angiogenic potential of albendazole suggests that the action of this anti-helminthic during *T. spiralis* infection is not confined to the structural damage of the parasite cuticle, but extends to affect the host immunopathological response, while still requiring an adjuvant such as MCS to synergize with its immune-mediated action.

## Data Availability

The data sets used and/or analyzed during the current study are available from the corresponding author on reasonable request.
